# Differences in Self-Esteem Between Cat Owners, Dog Owners, and Individuals Without Pets

**DOI:** 10.3389/fvets.2020.00552

**Published:** 2020-09-02

**Authors:** Claudia Schulz, Hans-Helmut König, André Hajek

**Affiliations:** Department of Health Economics and Health Services Research, University Medical Center Hamburg-Eppendorf, Hamburg, Germany

**Keywords:** self-esteem, animal ownership, pets, rosenberg scale, aging

## Abstract

**Introduction:** Pet ownership may provide an additional source of social support and may contribute to the owner's self-esteem. Self-esteem is considered a basic human need and is associated with psychological conditions such as depressive symptoms. To date, there is limited knowledge on the association between keeping a pet and self-esteem.

**Objectives:** The aim of this study was to determine whether cat owners, dog owners, and individuals without pets differ in terms of self-esteem (total sample and stratified by sex).

**Methods:** Data were taken from the German Aging Survey (wave 5; nationally representative sample of individuals residing in private households ≥40 years). In this survey, the widely used and well-established Rosenberg scale was used to quantify self-esteem. Socioeconomic, lifestyle, and health-related factors were adjusted for in the regression analysis (*n* = 5,485).

**Results:** Multiple linear regressions showed that dog owners reported higher self-esteem scores compared to individuals without pets (β = 0.04, *p* < 0.05). Similarly, male dog owners reported higher self-esteem scores compared to men without pets (β = 0.07, *p* < 0.01). In contrast, female cat owners reported lower self-esteem scores compared to women without pets (β = −0.07, *p* < 0.01).

**Conclusion:** Study findings showed a link between owning a cat and lower self-esteem (women), as well as between owning a dog and higher self-esteem (total sample; men). Future studies should concentrate on investigating the underlying mechanisms. Furthermore, longitudinal studies are needed to better understand the link between animal ownership and self-esteem.

## Introduction

In Germany, as well as in numerous other countries, a demographic change in the population is expected, with a shift in the age structure from young to old (1). Due to the aging population, the number of individuals with advanced age will increase.

A considerable proportion of older individuals are widowed, divorced, or have stayed single, and this proportion increases in older age (2). Likewise, grown-up children may have moved out to live in a separate household, or even another city, decreasing the spatial proximity of older individuals to children and other kin. Therefore, older persons are at risk for loneliness, social isolation, and reduced health and well-being (3). Among other things, these factors can increase morbidity or mortality (4, 5).

According to the attachment theory by Bowlby (6), there is a human need to be attached to somebody and to maintain relationships and a need for a sense of belonging. In the case of social isolation, this human attachment could be substituted by a human–pet attachment. Keeping pets has been shown to correlate with positive health outcomes when facing serious health issues, e.g., a reduced risk for mortality after a heart attack (7). Furthermore, there is evidence that pet owners in late life may have better psychosocial health, such as less depressive symptoms or decreased loneliness (8–11).

More generally, pet ownership may play a significant role in people's lives. A pet may provide an additional source of social support by providing company and being part of the household (12, 13). There is evidence of numerous benefits of pet ownership, including satisfaction with life and happiness (14). Companionship provided by pet ownership may be particularly important for older people to alleviate social isolation. There is broad evidence supporting the association of social support with beneficial effects related to cardiovascular, endocrine, and immune functioning (15), as well as self-esteem (16). Therefore, pet ownership may also be associated with self-esteem. Self-esteem is considered a basic human need for, inter alia, the respect from others in form of recognition, success, and admiration (17). It is a significant predictor for psychological diseases, e.g., depression or anxiety (18). There is evidence that owning a pet may increase self-esteem for preadolescents and young adults (19–21). The question is whether this also applies to older adults.

There is evidence that males may score slightly higher on self-esteem than females (22, 23). These sex differences may also feature in the relationship with pet ownership. However, there is no literature on sex differences in the association between pet ownership and self-esteem. However, one study found that female cat owners had a higher depression score than male cat owners (24), while another study did not find interaction effects of gender for the link between pet ownership and loneliness (9). When distinguishing between dog owners and cat owners, further literature found that dog owners are less often socially isolated and depressed (11, 24).

To date, there is limited knowledge on the association between keeping a pet and self-esteem. There is one study showing that animal-assisted therapy may help to increase self-esteem for patients with depressive and psychiatric disorders (25). Another study found that pet owners tend to have higher self-esteem than non-owners in general (13). However, there is no study focusing on older people, who may be particularly vulnerable to low self-esteem, due to a possible lack of social support. The question arises whether pets may contribute to owner's self-esteem by providing meaningful social support, and whether people of both sexes benefit from pet ownership to the same degree. Also, there may be differences between cats and dogs as most popular companion animals. Thus, the objective of this study was to determine whether older cat owners, dog owners, and individuals without pets differ in terms of self-esteem (total sample and stratified by sex). To investigate this, we used a large survey of individuals residing in Germany aged 40 and over in order to provide nationally representative findings. Knowledge on a link between owning a pet and self-esteem may particularly be helpful to address individuals who score low in self-esteem.

## Materials and Methods

### Sample

In our analysis, we used data from the fifth wave of the German Aging Survey (DEAS), a nationally representative study of non-institutionalized middle-aged and older adults (40+). It has a cohort-sequential design and is funded by the Federal Ministry for Family Affairs, Senior Citizens, Women and Youth (BMFSFJ). The Institute for Applied Social Sciences (infas) conducted the fieldwork for all waves.

Cross-sectional samples were drawn in 1996 (first wave), 2002 (second wave), 2008 (third wave), and 2014 (fifth wave), whereas the fourth wave was a pure panel sample (i.e., only including individuals who had already taken part before). The samples have been disproportionally stratified (by age, region, and sex). It is worth noting that the data relate to different households. In 2014, the response rate was 25% for first-time participants (resulting in more than 6,000 participants who participated for the first time in 2014). The response rate was 61% for participants who had already been interviewed previously (resulting in more than 4,000 individuals who participated again in 2014). The response rates of the DEAS study are in line with other German studies (26). After the interview (covering general topics such as sex or age), individuals were asked to fill out a drop-off questionnaire, which included questions that are more sensitive (e.g., regarding self-esteem). In 2014, a total of 7,952 individuals took part in the interview and additionally filled out the drop-off questionnaire. Moreover, we further restricted our sample to individuals with at least one child (who filled out the drop-off questionnaire), resulting in 6,927 individuals. We restricted our sample to individuals with one or more children because we included the distance to the nearest residential living child as a covariate in our regression model. Due to missing observations in regression, our analytical sample equaled 5,485 observations. More details regarding the DEAS study have been provided by Klaus et al. (27).

All participants provided written informed consent. As the criteria for an ethical statement were not fulfilled (such as risk for the respondents or use of invasive methods), ethics committee approval was not required.

### Outcome Measures

In the drop-off questionnaire, the Rosenberg scale was used to assess general self-esteem. It measures the worth dimension of self-esteem. Sample items include “I feel that I have a number of good qualities,” “I feel I do not have much to be proud of,” “At times I think I am no good at all,” or “I take a positive attitude toward myself.” It is a widely accepted and rigorously tested psychometric assessment focused on measuring self-esteem (28). This scale consists of 10 items (in each case: 1 = strongly agree to 4 = strongly disagree). Five items have been recoded (“On the whole, I am satisfied with myself”; “I am able to do things as well as most other people”; “I feel that I have a number of good qualities”; “I feel that I‘m a person of worth, at least on an equal plane with others”; “I take a positive attitude toward myself”). In line with accepted scoring for Rosenberg's scale, values were ascertained by averaging the items, with higher values indicating higher self-esteem (range: 1–4, Cronbach's alpha was 0.82 in our study).

### Independent Variables

Our main independent variable was pet ownership (no; yes). Individuals owning one or more pet/s were subsequently asked whether they own one or more cat/s, one or more dog/s, and other pet/s (multiple responses were possible). We decided to exclude the category “other pet/s” because of its unclear nature. More specifically, we defined cat owners as individuals who own one or more cat/s but do not own any other pets. In the same vein, dog owners were defined as individuals who own one or more dog/s but do not own any other pets.

As covariates, we included socioeconomic (age, family status, income poverty, and the distance to the nearest residential living), lifestyle (body mass index, smoking status, alcohol consumption, and the frequency of sports activities) and health-related variables (self-rated health, physical functioning, and the number of physical illnesses). Family status was measured by a set of dummy variables (married, living together with spouse; married, living separated from spouse; single; divorced; widowed). Income poverty was assessed using the threshold of 60% of median net-equivalence income. This is based on the OECD modified equivalence scale. The distance to the nearest residential living child (in the same household; in the same house (other household); in the neighborhood; in the same town; another town in Germany, but can be reached within 2 h; farther away, in Germany; farther away, abroad) was also used in the regression analysis. Furthermore, regarding lifestyle factors, the body mass index [BMI; defined as weight (kg) divided by height squared (m^2^)], smoking status (non-smoker; former smoker; casual smoker; daily smoker), alcohol consumption, and the frequency of sports activities (in both cases, categories were as follows: “never,” “rarer than once a month,” “one to three times a month,” “once a week,” “several times a week,” and “daily”) were used in the regression analysis. Regarding health-related covariates, physical functioning [subscale physical functioning of the SF-36 (29); from 0 (worst) to 100 (best)], self-rated health [ranging from very good (1) to very bad (5)], and the number of chronic diseases (e.g., bad circulation or diabetes; from 0 to 11) were used as covariates.

### Statistical Analysis

Stratified by owning a cat, owning a dog, and individuals without pets, sample characteristics were displayed using descriptive statistics. Subsequently, multiple linear regressions were computed to determine the link between owning a cat or dog and self-esteem (total sample; stratified by sex). Additionally, the analysis was stratified by age category (40–64 years; 65 years and older). The results were considered statistically significant when the *p* < 0.05. Stata 15.1 (StataCorp, College Station, Texas, USA) was used to perform statistical analyses.

## Results

### Sample Characteristics

Sample characteristics stratified by owning a cat, owning a dog, and individuals without pets are depicted in Table 1.

**Table 1:** Descriptive statistics of the study population.

	**Cat owners**		**Dog owners**		**Without pets**	
	**Women**	**Men**	**Women**	**Men**	**Women**	**Men**
Number of individuals: *N* (%)	445 (57.3%)	332 (42.7%)	257 (50.1%)	256 (49.9%)	2,078 (49.5%)	2,117 (50.5%)
Age in years: Mean (SD)	62.0 (±10.8)	62.1 (±10.2)	60.6 (±9.7)	62.1 (±9.5)	65.4 (±10.7)	67.7 (±10.8)
**Marital status:** ***N*** **(%)**						
- Married, living together with spouse	289 (64.9%)	278 (83.7%)	171 (66.5%)	227 (88.7%)	1,379 (66.4%)	1,705 (80.5%)
- Married, living separated from spouse	14 (3.2%)	5 (1.5%)	6 (2.3%)	3 (1.2%)	27 (1.3%)	37 (1.8%)
- Widowed	72 (16.2%)	29 (8.7%)	32 (12.5%)	14 (5.5%)	234 (11.3%)	155 (7.3%)
- Divorced	61 (13.7%)	12 (3.6%)	39 (15.2%)	8 (3.1%)	382 (18.4%)	183 (8.6%)
- Single	9 (2.0%)	8 (2.4%)	9 (3.5%)	4 (1.6%)	56 (2.7%)	37 (1.8%)
**Distance to the nearest residential living child:** ***N*** **(%)**						
- In the same household	141 (31.7%)	110 (33.1%)	78 (30.4%)	93 (36.3%)	363 (17.5%)	391 (18.5%)
- In the same house (other household)	41 (9.2%)	23 (6.9%)	16 (6.2%)	18 (7.0%)	139 (6.7%)	138 (6.5%)
- In the neighborhood	61 (13.7%)	35 (10.5%)	25 (9.7%)	18 (7.0%)	230 (11.1%)	243 (11.5%)
- In the same town	58 (13.0%)	48 (14.5%)	38 (14.8%)	41 (16.0%)	498 (24.0%)	458 (21.6%)
- Another town in Germany, but it can be reached within 2 h	112 (25.2%)	88 (26.5%)	77 (30.0%)	67 (26.2%)	623 (30.0%)	685 (23.4%)
- Farther away, in Germany	23 (5.2%)	27 (8.1%)	22 (8.6%)	18 (7.0%)	189 (9.1%)	181 (8.6%)
- Farther away, abroad	9 (2.0%)	1 (0.3%)	1 (0.4%)	1 (0.4%)	36 (1.7%)	21 (1.0%)
**Income poverty:** ***N*** **(%)**						
- Presence of income poverty	59 (13.3%)	25 (7.5%)	26 (10.1%)	25 (9.8%)	198 (9.5%)	147 (6.9%)
- Absence of income poverty	386 (86.7%)	307 (92.5%)	231 (89.9%)	231 (90.2%)	1,880 (90.5%)	1,970 (93.1%)
Body mass index: mean (SD)	26.8 (±4.9)	27.9 (±4.6)	26.6 (±4.8)	27.4 (±3.9)	26.4 (±4,8)	27.3 (±4.0)
**Smoking behavior:** ***N*** **(%)**						
- Daily smoker	71 (16.0%)	53 (16.0%)	51 (19.8%)	55 (21.5%)	227 (10.9%)	247 (11.7%)
- Casual smoker	8 (1.8%)	14 (4.2%)	11 (4.3%)	15 (5.9%)	65 (3.1%)	94 (4.4%)
- Former smoker	135 (30.3%)	152 (45.8%)	92 (35.8%)	122 (47.7%)	567 (27.3%)	992 (46.9%)
- Non-smoker	231 (51.9%)	113 (34.0%)	103 (40.1%)	64 (25.0%)	1,219 (58.7%)	784 (37.0%)
**Physical activity:** ***N*** **(%)**						
- Daily	41 (9.2%)	18 (5.4%)	24 (9.3%)	19 (7.4%)	184 (8.9%)	184 (8.7%)
- Multiple times a week	117 (26.3%)	94 (28.3%)	64 (24.9%)	51 (19.9%)	617 (29.7%)	568 (26.8%)
- Once a week	87 (19.6%)	59 (17.8%)	57 (22.2%)	36 (14.1%)	448 (21.6%)	346 (16.3%)
- One to three times a month	29 (6.5%)	32 (9.6%)	16 (6.2%)	28 (10.9%)	134 (6.5%)	174 (8.2%)
- Less frequently	52 (11.7%)	45 (13.6%)	27 (10.5%)	49 (19.1%)	184 (8.9%)	261 (12.3%)
- Never	119 (26.7%)	84 (25.3%)	69 (26.85%)	73 (28.5%)	511 (24.6%)	584 (27.6%)
**Alcohol intake:** ***N*** **(%)**						
- Daily	22 (4.9%)	71 (21.4%)	12 (4.7%)	51 (19.9%)	129 (6.2%)	392 (18.5%)
- Multiple times a week	64 (14.4%)	118 (35.5%)	46 (17.9%)	67 (26.2%)	381 (18.3%)	676 (31.9%)
- Once a week	66 (14.8%)	49 (14.8%)	35 (13.6%)	41 (16.0%)	331 (15.9%)	345 (16.3%)
- One to three times a month	68 (15.3%)	28 (8.4%)	38 (14.8%)	27 (10.6%)	300 (14.4%)	209 (9.9%)
- Less frequently	145 (32.6%)	44 (13.3%)	80 (31.1%)	42 (16.4%)	677 (32.6%)	312 (14.7%)
- Never	80 (18.0%)	22 (6.6%)	46 (17.9%)	28 (10.9%)	260 (12.5%)	183 (8.6%)
Self-rated health (1 = very good to 5 = very bad): mean (SD)	2.5 (±0.8)	2.6 (±0.9)	2.5 (±0.9)	2.6 (±0.9)	2.5 (±0.8)	2.5 (±0,8)
Number of physical illnesses (from 0 to 11): mean (SD)	2.6 (±1.9)	2.6 (±1.8)	2.5 (±1.9)	2.7 (±2.0)	2.6 (±1.9)	2.7 (±1.9)
Physical functioning [from 0 (worst) to 100 (best)]: mean (SD)	81.1 (±23.2)	82.2 (±21.9)	80.1 (±25.0)	83.0 (±21.6)	79.3 (±24.4)	83.6 (±21.4)
Self-esteem: mean (SD)	3.4 (±0.4)	3.4 (±0.4)	3.4 (±0.4)	3.4 (±0.4)	3.4 (±0.4)	3.4 (±0.4)

In the total sample, mean age was 65.4 years (standard deviation (SD) 10.9), and 50.7% were female. Of cat owners, 57.3% were female. 50.1% of dog owners were female, and 49.5% of non-owners were female (Table 1). Among all females in the dataset, 16.0% owned cats, 9.2% owned dogs, and 74.7% owned no pets. Among all males, 12.3% owned cats, 9.5% owned dogs, and 78.3% owned no pets (results not shown). Mean self-esteem was 3.4 (SD 0.4) among dog owners, cat owners, and non-owners, both female and male. Further details are reported in Table 1.

### Regression Analysis

The results of the multiple linear regressions are displayed in Table 2. For the total sample, cat owners had marginally significant lower self-esteem (β = −0.03, *p* < 0.1) than individuals without pets, whereas dog owners had statistically significant higher self-esteem than individuals without pets (β = 0.04, *p* < 0.05). Stratification by sex showed that these results were driven by either males or females: female cat owners had significant decreased self-esteem (β = −0.07, *p* < 0.01), and male dog owners had significant increased self-esteem (β = 0.07, *p* < 0.01), compared to non-owners, respectively.

**Table 2:** Determinants of self-esteem.

	**(1)**	**(2)**	**(3)**
	**Self-esteem—total sample**	**Self-esteem—men**	**Self-esteem—women**
Potential confounders^a^	✓	✓	✓
Pet ownership: - owning a cat (ref.: not owning a pet)	−0.03+	0.02	−0.07^**^
	(0.02)	(0.02)	(0.02)
- Owning a dog	0.04^*^	0.07^**^	−0.00
	(0.02)	(0.02)	(0.03)
Constant	3.45^***^	3.26^***^	3.52^***^
	(0.07)	(0.10)	(0.10)
Individuals	5,485	2,705	2,780
*R*^2^	0.13	0.16	0.12

*beta-coefficients are reported; robust standard errors in parentheses*.

*** p < 0.001,

** p < 0.01,

* *p < 0.05, + p < 0.10. Observations with missing values were dropped (listwise deletion)*.

a *Potential confounders include age, marital status, distance to the nearest residential living child, income poverty, body mass index, smoking behavior, alcohol intake, frequency of sports activities, self-rated health, physical functioning, and the number of physical illnesses*.

The analysis was further separated by age which showed that the main driver of these results were individuals aged 65 and more years (Table 3). Among those aged 65 years and over, both men and women who own a dog showed statistically significant increased self-esteem (β = 0.07, *p* < 0.05), compared to individuals without pets. When separating those aged 65 years and over by sex, female cat owners had significant decreased self-esteem (β = −0.07, *p* < 0.05) and male dog owners had increased self-esteem, which was, however, no longer significant (β = 0.07, *p* < 0.1). For individuals aged 40–64 years, cat (β =^.^-0.03) and dog (β = 0.01) owners did not show significant results, compared to non-owners. Male pet owners aged 40–64 years had non-significant or marginally significant increased self-esteem (cat owners: β = 0.01; dog owners: β = 0.05; *p* < 0.1), whereas female pet owners aged 40–64 years had non-significant or marginally significant decreased self-esteem (cat owners: β = −0.06; *p* < 0.1; dog owners: β = −0.04).

**Table 3:** Determinants of self-esteem—additionally stratified by age (40–64 years; 65 years and over).

	**40–64**			**65+**		
	**(1)**	**(2)**	**(3)**	**(4)**	**(2)**	**(3)**
	**Self-esteem—total**	**Self-esteem—**	**Self-esteem—**	**Self-esteem—**	**Self-esteem—**	**Self-esteem—**
	**sample**	**men**	**women**	**total sample**	**men**	**women**
Potential confounders^a^	✓	✓	✓			
Pet ownership: - owning a cat (ref.: not owning a pet)	−0.03	0.01	−0.06+	−0.03	0.02	−0.07^*^
	(0.02)	(0.03)	(0.03)	(0.02)	(0.03)	(0.03)
- Owning a dog	0.01	0.05+	−0.04	0.07^*^	0.07+	0.06
	(0.02)	(0.03)	(0.03)	(0.03)	(0.04)	(0.04)
Constant	3.35^***^	3.18^***^	3.45^***^	3.46^***^	3.29^***^	3.48^***^
	(0.12)	(0.17)	(0.17)	(0.13)	(0.18)	(0.21)
Individuals	2,521	1,122	1,399	2,964	1,583	1,381
*R*^2^	0.18	0.22	0.17	0.10	0.12	0.10

*Comments: beta-coefficients are reported; robust standard errors in parentheses*.

*** p < 0.001,

* *p < 0.05, +p < 0.10. Observations with missing values were dropped (listwise deletion)*.

a *Potential confounders include age, marital status, distance to the nearest residential living child, income poverty, body mass index, smoking behavior, alcohol intake, frequency of sports activities, self-rated health, physical functioning, and the number of physical illnesses*.

Across all models, significant decreased self-esteem was associated with an increasing number of chronic diseases, decreasing self-rated health, and the presence of income poverty.

## Discussion

### Main Findings

Dog owners reported statistically significant higher self-esteem scores compared to individuals without pets and cat owners reported marginally significant lower self-esteem scores compared to individuals without pets (Table 2). Male dog owners reported significant higher self-esteem scores compared to men without pets. In contrast, female cat owners reported significant lower self-esteem scores compared to women without pets. These findings were driven by older individuals aged 65 years and over (Table 3).

### Relation to Previous Studies and Possible Explanations

Several explanations for the relationship between pet ownership and self-esteem are possible. First, the reaction of others, such as friends and family, colleagues and even strangers, can affect the self-esteem of the pet owner (30). If pet ownership is associated with a positive image, the owner will receive positive feedback when, e.g., they are seen with their pet or show pictures of it.

Second, the behavior of pets may play a role. A dog may consider his owner as a “pack” member with both striving for cohesiveness and appeasement. The owner may consider himself respected by the dog and being part of a cohort which may improve his self-image. A cat on the other hand is not a pack animal by nature. Therefore, it usually does not treat its owner as a team member. A cat tends to behave in a more self-determined and independent way, rather than being affectionate and devoted as a dog would be. Therefore, cat owners may not benefit from a “pack” or cohort membership due to their pet, as dog owners may do.

Furthermore, dogs may increase the owner's physical activity because of the dog's need for exercise, both for adolescents (31, 32) and persons in later life (10, 33, 34). This increases the opportunity for social encounters and interactions, e.g., when meeting other persons during a dog walk. Dogs are gregarious by nature and thus may force interactions with other people, e.g., other dog owners, with a common and easy topic of interest—dogs. Dog owners may develop a sense of belonging when taking their dog for a walk in a nearby dog park or exercise area, with other dog owners also living nearby, or when visiting a dog school. Doing these activities regularly in a nearby environment is a good opportunity for regularly meeting similar individuals and developing a positive relationship with them. In line with this argument, a recent study has shown that owning a dog is associated with a better relationship with neighbors (10). Social interactions may increase the chance for social support as well as attachment to others (e.g., neighbors), which would then increase the individual's self-esteem. Furthermore, there is evidence that individuals who are more physically active in general may have increased self-esteem (35, 36).

On the other hand, cats usually do not leave the owner's house or do it on their own without needing their owners to accompany them. Subsequently, a cat does not necessarily increase the chance for either physical activities or social interactions. Therefore, cat owners may not benefit in the same way that dog owners benefit from the gregarious characteristics a dog has, and the physical and social effects of walking the dog.

In our study, gender-stratified regressions revealed that female cat owners reported lower self-esteem scores compared to women without pets, and male dog owners reported higher self-esteem scores compared to men without pets. It may be hypothesized that an individual's sex, his or her choice of owning a dog or a cat, and his or her self-esteem may be related. Particularly, males may choose to own a dog, when longing for social or physical activity in general, or for maintaining the level of social or physical activity when getting older. This would be accompanied by increased self-esteem, as described above. Furthermore, men may be more drawn to dogs in order to be part of a pack or even be the pack leader, because it feeds a desire to be independent of other people (23). On the other hand, particularly older women who own a cat may suffer from the perceived (though dated) stigma of being an “old maid” (if they do not have a partner) or cat lady, stereotypes that suggest these women are supposedly prone to become disconnected from society (37–39). This, in turn may be associated with reduced self-esteem. However, future studies are required to confirm our assumptions.

There are few studies investigating pet ownership and self-esteem, which have focused on children or young adults (19–21, 40). They found that pet owners in general have a higher self-esteem than non-owners, in line with our results with respect to dog ownership. Another study investigated the general population and did not find significant differences between pet owners and non-owners, nor between sexes (14). The authors argued that owners and non-owners may be equal in terms of personality traits. However, pet owners may be considered by the majority of the population as a homogenous group with certain social stereotypes. For example, dog owners may be perceived as more active, livelier, and with a higher self-esteem, even if this is not true. This reaction of others may contribute to one's own self-esteem.

One study from Germany investigated older individuals aged 65 years and more and living alone, using the same dataset as our study. This study found that, compared to individuals without pets, dog owners were less socially isolated (11). Cat ownership did not provide any benefits regarding social isolation and loneliness. Similarly, one study investigating persons aged 65 years and over in Norway found higher depression scores and more negative self-rated health among cat owners than among both dog owners and non-owners (24). Another study based on in-depth interviews of pet owners aged 75 years and over living in Australia found that cat owners tend to be more socially isolated than dog owners (41). However, one study from the United States investigating persons aged 60 years and more found that cat owners reported significantly fewer depressive symptoms than dog owners (42).

It appears plausible that self-selection may explain our findings—at least to some degree. This means that men who score high in self-esteem may be more likely to buy a dog, or women who score low in self-esteem may be more likely to buy a cat (influenced by unobserved factors like personality characteristics). To tackle this issue, longitudinal studies that analyze the impact of buying a pet on self-esteem are needed. Furthermore, the effect sizes in our results were rather small. Thus, although the findings were statistically significant, the association between pet ownership, the owner's self-esteem, and his or her sex may not be very strong.

### Strengths and Limitations

As one of very few studies, we showed that differences in self-esteem exist between cat owners, dog owners, and individuals without pets. The widely used and well-established Rosenberg scale was used to measure self-esteem. For this study, data were taken from a large, nationally representative study. It should be noted that we restricted our sample to individuals with at least one child. While we were able to differentiate between cat and dog owners, upcoming studies should take into consideration other dimensions [such as the quality of the human–pet relationship (43)]. Moreover, there may be several unobserved confounders such as personality factors which should be included in future studies (given the data availability). Cross-sectional studies, like ours, have well-known and well-accepted limitations. For example, we cannot dismiss the possibility that the directionality goes from self-esteem to pet ownership (reverse causality). Future studies, for example based on longitudinal designs, are required to clarify the directionality. Klaus and Engstler (44) have shown that a small sample selection bias exists in the DEAS study.

## Conclusion

Study findings showed a link between owning a cat and lower self-esteem (for women), as well as between owning a dog and higher self-esteem (for the total sample and for men). Future studies should concentrate on investigating the underlying mechanisms. Furthermore, longitudinal studies are needed to better understand the link between animal ownership and self-esteem.

## Data Availability Statement

Publicly available datasets were analyzed in this study. The data used in this study are third party data. The anonymized data sets of the DEAS (1996, 2002, 2008, 2011, 2014, and 2017) are available for secondary analysis. The data has been made available to scientists at universities and research institutes exclusively for scientific purposes. The use of data is subject to written data protection agreements. Microdata of the German Aging Survey (DEAS) is available free of charge to scientific researchers for non-profitable purposes. The FDZ-DZA provides access and support to scholars interested in using DEAS for their research. However, for reasons of data protection, signing a data distribution contract is required before data can be obtained. Please see for further Information (data distribution contract): https://www.dza.de/en/fdz/accessto-data/formular-deas-en-english.html.

## Ethics Statement

Ethical review and approval was not required for the study on human participants in accordance with the local legislation and institutional requirements. Written informed consent for participation was not required for this study in accordance with the national legislation and the institutional requirements.

## Author Contributions

CS, H-HK, and AH collaborated in the design and concept of analyses, preparation of data, statistical analysis and interpretation of data, and preparing of the manuscript. All authors critically reviewed the manuscript, provided significant editing of the article, and approved the final manuscript.

## Conflict of Interest

The authors declare that the research was conducted in the absence of any commercial or financial relationships that could be construed as a potential conflict of interest.

## References

[1] Statistisches Bundesamt Deutschland Bevölkerung Deutschlands bis 2060:13. koordinierte Bevölkerungsvorausrechnung. Wiesbaden: Statistisches Bundesamt (2015).

[2] Statistisches Bundesamt Deutschland Genesis-Online Datenbank. (2019) Available online at: https://www-genesis.destatis.de/genesis/online (accessed January 16, 2020).

[3] Courtin E Knapp M. Social isolation, loneliness and health in old age: a scoping review. Health Soc Care Commun. (2017) 25:799–812. 10.1111/hsc.12311 26712585

[4] Iecovich E Jacobs JM Stessman J. Loneliness, social networks, and mortality: 18 years of follow-up. Int J Aging Human Dev. (2011) 72:243–63. 10.2190/AG.72.3.e 21834390

[5] Pantell M Rehkopf D Jutte D Syme SL Balmes J Adler N. Social isolation: a predictor of mortality comparable to traditional clinical risk factors. Am J Public Health. (2013) 103:2056–62. 10.2105/AJPH.2013.301261 24028260 PMC3871270

[6] Bowlby J. The making breaking of affectional bonds: II. Some principles of psychotherapy: the fiftieth maudsley lecture (expanded version). Br J Psychiatry. (1977) 130:421–31. 10.1192/bjp.130.5.421 861423

[7] Friedmann E Thomas SA. Pet ownership, social support, and one-year survival after acute myocardial infarction in the Cardiac Arrhythmia Suppression Trial (CAST). Am J Cardiol. (1995) 76:1213–7. 10.1016/S0002-9149(99)80343-9 7502998

[8] Cheung C-k Kam PK. Conditions for pets to prevent depression in older adults. Aging Mental Health. (2018) 22:1627–33. 10.1080/13607863.2017.1385723 28976782

[9] Stanley IH Conwell Y Bowen C Van Orden KA. Pet ownership may attenuate loneliness among older adult primary care patients who live alone. Aging Mental Health. (2014) 18:394–9. 10.1080/13607863.2013.837147 24047314 PMC3944143

[10] Taniguchi Y Seino S Nishi M Tomine Y Tanaka I Yokoyama Y. Physical, social, and psychological characteristics of community-dwelling elderly Japanese dog and cat owners. PLoS ONE. (2018) 13:e0206399. 10.1371/journal.pone.0206399 30427858 PMC6241120

[11] Hajek A König H-H. How do cat owners, dog owners and individuals without pets differ in terms of psychosocial outcomes among individuals in old age without a partner? Aging Mental Health. (2019). 10.1080/13607863.2019.1647137. [Epub ahead of print]. 31364868

[12] McNicholas J Gilbey A Rennie A Ahmedzai S Dono J-A Ormerod E. Pet ownership and human health: a brief review of evidence and issues. BMJ. (2005) 331:1252–4. 10.1136/bmj.331.7527.1252 16308387 PMC1289326

[13] McConnell A Brown C. Friends with benefits: on the positive consequences of pet ownership. J Personal Soc Psychol. (2011) 101:1239–52. 10.1037/a0024506 21728449

[14] Johnson SB Rule WR Personality characteristics and self-esteem in pet owners and non-owners. Int J Psychol. (1991) 26:241–52. 10.1080/00207599108247889

[15] Uchino BN Cacioppo JT Kiecolt-Glaser JK. The relationship between social support and physiological processes: a review with emphasis on underlying mechanisms and implications for health. Psychol Bulletin. (1996) 119:488. 10.1037/0033-2909.119.3.488 8668748

[16] Harter S The development of self-representations during childhood and adolescence. In: Leary MR Tangney JP, editors. Handbook of Self and Identity. New York, NY: The Guilford Press (2003), p. 610–42.

[17] Maslow AH Frager R Fadiman J McReynolds C Cox R Motivation and Personality (3rd). New York, NY: Pearson Longman (1987).

[18] Sowislo JF Orth U. Does low self-esteem predict depression and anxiety? A meta-analysis of longitudinal studies. Psychol Bulletin. (2013) 139:213. 10.1037/a0028931 22730921

[19] Angle RL Utilization of the Pet Bonding Scale to Examine the Relation Between the Human/Companion Animal Bond and Self-Esteem in Pre-Adolescence. Houston, TX: University of Houston (1995).

[20] Hyde KR Kurdek L Larson PC Relationships between pet ownership and self-esteem, social sensitivity, interpersonal trust. Psychol Rep. 52:110 (1983) 10.2466/pr0.1983.52.1.110

[21] Bierer RE The Relationship Between Pet Bonding, Self-Esteem, and Empathy in Preadolescents. New Mexico: University of New Mexico (2001).

[22] Kling KC Hyde JS Showers CJ Buswell BN. Gender differences in self-esteem: a meta-analysis. Psychol Bulletin. (1999) 125:470. 10.1037/0033-2909.125.4.470 10414226

[23] Josephs RA Markus HR Tafarodi RW. Gender and self-esteem. J Personal Soc Psychol. (1992) 63:391. 10.1037/0022-3514.63.3.391 1403622

[24] Enmarker I Hellzén O Ekker K Berg AGT Depression in older cat and dog owners: the Nord-Trøndelag Health Study (HUNT)-3. Aging Mental Health. (2015) 19:347–52. 10.1080/13607863.2014.933310 24990174

[25] Peluso S De Rosa A De Lucia N Antenora A Illario M Esposito M. Animal-Assisted therapy in elderly patients: evidence and controversies in dementia and psychiatric disorders and future perspectives in other neurological diseases. J Geriatr Psychiatry Neurol. (2018) 31:149–57. 10.1177/0891988718774634 29764282

[26] Neller K Kooperation und verweigerung. eine non-response-studie. ZUMA Nachrichten. (2005). 29:9–36.

[27] Klaus D Engstler H Mahne K Wolff JK Simonson J Wurm S. Cohort profile: the German Ageing Survey (DEAS). Int J Epidemiol. (2017) 46:1105. 10.1093/ije/dyw326 28180273 PMC5837219

[28] Ferring D Filipp S-H Messung des selbstwertgefühls: befunde zu reliabilität, validität und stabilität der rosenberg-skala. Diagnostica. (1996) 42:284–92.

[29] Ware JE Jr Sherbourne CD. The MOS 36-item short-form health survey (SF-36): I. Conceptual framework and item selection. Med care. (1992) 30:473–83. 10.1097/00005650-199206000-00002 1593914

[30] Argyle M Social Encounters: Contributions to Social Interaction. New York, NY: Routledge (2017) 10.4324/9781315129501

[31] Sirard JR Patnode CD Hearst MO Laska MN. Dog ownership and adolescent physical activity. Am J Prevent Med. (2011) 40:334–7. 10.1016/j.amepre.2010.11.007 21335266 PMC3395162

[32] Yabroff K Trojano R Berrigan D. Walking the dog: is pet ownership associated with physical activity in California? J Phys Activ Health. (2008) 5:13. 10.1123/jpah.5.2.216 18382031

[33] Feng Z Dibben C Witham M Donnan P Vadiveloo T Sniehotta F. Dog ownership and physical activity in later life: A cross-sectional observational study. Prevent Med. (2014) 66:101–6. 10.1016/j.ypmed.2014.06.004 24931433

[34] Shibata A Oka K Inoue S Christian H Kitabatake Y Shimomitsu T. Physical activity of Japanese older adults who own and walk dogs. Am J Prevent Med. (2012) 43:5. 10.1016/j.amepre.2012.06.019 22992362

[35] McAuley E Blissmer B Katula J Duncan TE Mihalko SL Physical activity, self-esteem, and self-efficacy relationships in older adults: a randomized controlled trial. Ann Behav Med. (2000) 22:131 10.1007/BF02895777 10962706

[36] Sonstroem RJ Morgan WP. Exercise and self-esteem: rationale and model. Med Sci Sports Exercise. (1989) 21:329–37. 10.1249/00005768-198906000-00018 2659918

[37] Probyn-Rapsey F The “crazy cat lady”. In: Gruen L Probyn-Rapsey F, editors. Animaladies: Gender, Animals, Madness. New York, NY: Bloomsbury Publishing (2018). p. 175 10.5040/9781501342189.ch-012

[38] Lahad K A Table for One: A Critical Reading of Singlehood, Gender and Time. Manchester: Manchester University Press (2017). 10.2307/j.ctt1wn0s66

[39] Lahad K Hazan H The terror of the single old maid: on the insolubility of a cultural category. Women Stud Int Forum. (2014) 47:127–36. 10.1016/j.wsif.2014.08.001

[40] McConnell A Pet ownership and children's self-esteem in the context of war. J Personal Soc Psychol. (2011). 101:14 10.2752/089279399787000101

[41] Wells Y Rodi H Effects of pet ownership on the health and well-being of older people. Austr J Ageing. (2000) 19:143–8. 10.1111/j.1741-6612.2000.tb00167.x

[42] Branson SM Boss L Cron S Turner DC Depression, loneliness, and pet attachment in homebound older adult cat and dog owners. J Mind Med Sci. (2017) 4:38–48. 10.22543/7674.41.P3848

[43] Hoffman CL Chen P Serpell JA Jacobson KC. Do dog behavioral characteristics predict the quality of the relationship between dogs and their owners? Hum Anim Interact Bull. (2013) 1:20–37. 10.1037/e565452013-003 25685855 PMC4326091

[44] Klaus D Engstler H Daten und methoden des deutschen alterssurveys. In: Mahne K Wolff JK Simonson J Tesch-Römer C, editors. Altern im Wandel: Zwei Jahrzehnte Deutscher Alterssurvey (DEAS). Berlin (2016). p. 25–42. 10.1007/978-3-658-12502-8_2

